# Mobile APP-assisted family physician program for improving blood pressure outcome in hypertensive patients

**DOI:** 10.1186/s12875-023-01965-2

**Published:** 2023-01-10

**Authors:** Fang Xing, Yijia Guo, Nan Xia, Suolei Zhang, Jinfeng Yin, Liyi Qin, Chendi Zhu, Qing Gao, Junnan Jia, Yuesong Zhao, Yousheng Qi, Weimin Li

**Affiliations:** 1 Heyi Community Health Service Center, Fengtai District, No. 6, 2Nd Quarter, Heyidongli, Beijing, 100076 China; 2 Beijing Chest Hospital, Capital Medical University, No. 9, Beiguan Street, Tongzhou District, Beijing, 101149 China; 3 Beijing Municipal Key Laboratory of Clinical Epidemiology, School of Public Health, Capital Medical University, Beijing, China; 4 National Tuberculosis Clinical Lab of China, Beijing Tuberculosis and Thoracic Tumour Research Institute and Beijing Key Laboratory in Drug Resistance Tuberculosis Research, Beijing, China; 5 Beijing Center for Disease Prevention and Control, Beijing, China; 6 Graduate School of Peking Union Medical College, Chinese Academy of Medical Science, Beijing, China

**Keywords:** Hypertension, Community and family medicine, Cohort study

## Abstract

**Background:**

This study was aimed to examine the effectiveness of App-assisted self-care in a Beijing community based on intelligent family physician-optimised collaborative model (IFOCM) program.

**Methods:**

We conducted a survey of 12,050 hypertensive patients between Jan 2014 and Dec 2021. Generalized linear model was used to analyze the covariates that associated with blood pressure (BP) control. Decision tree and random forest algorithm was used to extract the important factors of BP outcome.

**Results:**

The study included 5937 patients, mean age 66.2 ± 10.8, with hypertension in the baseline; 3108(52.4) were female. The community management resulted in mean systolic BP and diastolic BP reductions of 4.6 mmHg and 3.8 mmHg at follow-up. There were 3661 (61.6%) hypertension patients with BP control, increasing from 55.0% in 2014 to 75.0% in 2021. After adjusted for covariates, antihypertensive medication adherence, diabetes, and APP-assisted self-care were common predictors associated with BP control in GLM model and machine learning algorithm.

**Conclusion:**

Community management based on IFOCM program significantly improved BP control in hypertensive patients. APP-assisted self-care would be beneficial for the management of chronic disease.

**Supplementary Information:**

The online version contains supplementary material available at 10.1186/s12875-023-01965-2.

## Background

Hypertension has become one of the most important causes of disease burden in the world [1]. It is the leading risk factor of cardiovascular disease and stroke, accounting for nearly half of the morbidity and mortality [2–4]. A population-based study involved 1.7 million individuals revealed that hypertension awareness of 36%, treatment of 22.9% and control of only 5.7% in China [5]. Despite the hypertension control rate rising from 6.1% to 16.8% in recent years [6], that current management are insufficient to address the burden of hypertension [7, 8].

Self-management of blood pressure (BP), where patients management their own BP usually in a home environment, is an increasingly strategy of hypertension management [9, 10], which have been demonstrated to improve BP control in many Western countries [11–13]. The evidence for hypertension self-management is limited in China. Several studies [14–16] reported self-management may be a feasible and cost-effectiveness strategy for BP control in Chinese population. However, these studies were small sample size [14–16], cross-sectional [14] or intervention design [15], could not examine the effectiveness of community healthcare in controlling BP in the real world.

**Fig. 1:**
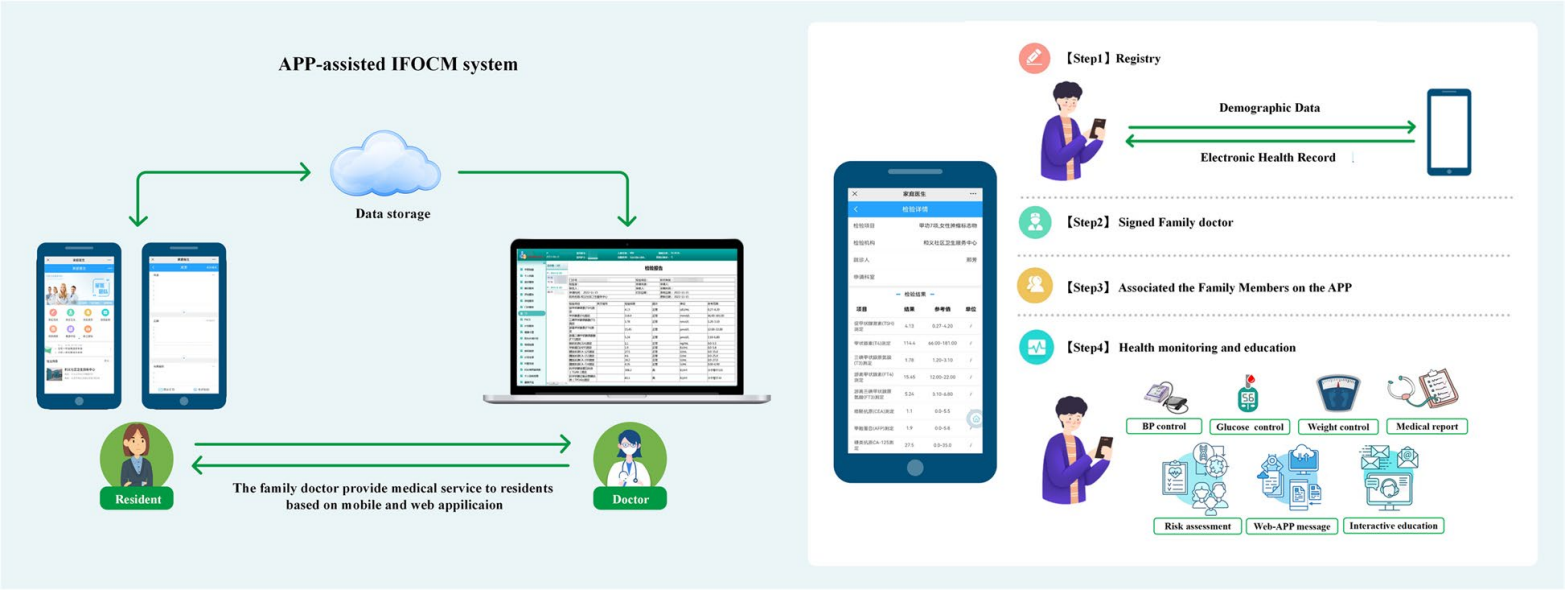
Overview of the APP-assisted IFOCM software system

When facing such large number of hypertensive patients, Chinese government launched the Basic Public Health Service Program in 2009 and Family Physician Program in 2016. Beijing was the earliest pilot city implementing the intelligent family physician-optimised coordination model (IFOCM) program in China [17]. With the popular of mobile health, smartphone also provides a promising approach access to self-care management in daily life. Our community health service center is one of the first facilities to apply APP-assisted self-care in the IFOCM program. APP-assisted IFOCM system is designed to help users make consistent monitor and management of physiological indicators, and remind them to take medicine or modify lifestyle. This software system includes an app for users and a web application for contracted family doctors (Fig. 1). In this study, we aimed to examine the effectiveness of BP control in our community and to investigate the role of mobile APP-assisted self-care in the present community-based cohort study.

## Methods

### Study design

This study was a community-based survey for patients with hypertension, performed by our community healthcare from Jan 2014 to December 2021. All patients having baseline record in the database of Beijing Primary Medical and Public Health Information System were initially considered for inclusion. Patients were included if they: (1) 18 years or older and diagnosed as having hypertension in the baseline; (2) had at least one follow-up record documented in the database; and (3) were not involved in other public health intervention program. Patients were excluded if they had severe neurological or psychiatric disorders; physical disability; and pregnancy hypertension. Informed consent from all subjects and/or their legal guardian was signed in the community health service center, and analysis was performed using deidentified data. The study was approved by the ethics committee of our community health service center. 

### Data collection

Deidentified data were extracted from the database, which included all patients with chronic diseases managed by community health service center. All individuals participated in baseline survey, physical examination, blood tests. The survey investigated the demographic characteristic, comorbidities such as hypertension, diabetes mellitus, coronary artery disease, and stroke. Physical examination included blood pressure, body weight, body height, waist girth, and hip girth. Blood tests assessed fasting blood-glucose and total cholesterol level. For residents participated IFOCM program, follow-up was conducted by team member in community. Follow-up information, such as systolic blood pressure (SBP), diastolic blood pressure (DBP), blood-glucose, blood lipid level, antihypertensive medication adherence, adverse drug reaction and follow-up date was recorded.

### Definition of variable and outcome

Hypertension was diagnosed as a maintained SBP/DBP ≥ 140/90 mmHg, or receiving antihypertensive medications. Baseline and follow-up body mass index (BMI, kg/m^2^) was calculated as the weight (kg) divided by squared height (m), and obesity was defined as BMI ≥ 27 at follow-up. Waist-hip ratio was calculated as the waist girth divided by hip girth, the ratio ≥ 0.9 was defined as increased level. The follow-up time was calculated as the last recorded visit date subtract baseline date.

We set two primary outcomes: BP control levels (continuous outcome) and BP control rates (categorical outcome). When treated BP as categorical outcome, we assessed the rates of patients with hypertension in control, which was according to the following definitions based on Chinese guideline [6]. For hypertension, BP control during a given visit as a SBP < 140 mmHg and DBP ≤ 90 mmHg, and for diabetes or coronary heart disease, a SBP ≤ 130 mmHg and DBP ≤ 80 mmHg.

### Machine learning

We used the random forest (RF) algorithm for the prediction of poor BP control of hypertension patients. RF is a machine learning algorithm that aggregates many predictions to reduce the variance and improve the robustness and precision of outputs [18]. A remarkable characteristic of the RF is that it offers an internal measure to show the importance of each variable on the prediction. Even if the data are missing or unbalanced [19], RF model can work very well for any type of problem regardless of sample size. Generally speaking, RF can quantify which variable contribute most to classification accuracy and suggests an important associated variables evaluated by the model, which can be optimized to obtain the best results from the data they are analyzing. In this study, we compared RF and other machine learning algorithms, including decision tree (DT), support vector machine(SVM), and naïve Bayes(NB) for predicting the outcome of BP control.

### Statistical analyses

The statistical analysis was performed by Stata (v.16.0) and R software (v.3.6.3). For normally distributed continuous variables, the values are expressed as mean ± standard deviation (SD) and compared using a 2-sided t-test. The categorical variables are expressed as frequencies, and comparisons are made using the Chi-squared test or Fisher’s exact test when appropriate.

To analyze the outcome of BP control levels, we estimated covariates that affect the values of SBP and DBP, a generalized linear model (GLM) was used to adjust for covariates including demographic characteristic, comorbidity, BMI, waist-hip ratio, baseline blood pressure, blood-glucose and total cholesterol level. Decreases in SBP and DBP in follow-up compared with baseline were considered dependent variables, whereas other covariates were considered independent variables. To analyze the outcome of BP control rates, we estimated covariates that associated with BP control in the GLM, poor BP control was considered dependent variable, and other similar covariates considered as independent variables were adjusted. Predictors of poor BP control manifested a statistical significance of P < 0.05 were subsequently utilized for different machine learning algorithm. Mean Decrease Gini (MDG) involved in RF algorithm with cross-validation was used to rank the important covariates with poor BP control.

## Results

### Baseline characteristics

A total of 44,039 community residents were recorded in the database at the baseline. The community center has 12,050 hypertensive patients, accounting for 27.3% of the population (Supplement Fig. 1). The difference between hypertensive patients with and without IFOCM management is shown in Supplement Table. Finally, there were 5937 participants thus included in the analysis of BP control, the mean age was 66.2 ± 10.8; 3108(52.4) were female. Baseline characteristics between participants with and without APP-assisted self-care is shown in Table 1. Compared to patients without APP-assisted self-care, APP-assisted group showed significantly greater age (66.5 ± 10.2 vs. 65.5 ± 12.4 years, *P* = 0.004), BMI (25.9 ± 3.5 vs. 25.6 ± 3.6 kg/m^2^, *P* = 0.052), waist (88.8 ± 9.5 vs. 87.6 ± 9.2 cm, *P* < 0.001) and hip (98.4 ± 9.2 vs. 96.9 ± 9.2 cm, *P* < 0.001); with significantly lower baseline SBP (128.0 ± 7.3 vs 130.8 ± 6.6 mmHg, *P* < 0.001) and DBP (76.8 ± 5.6 vs 77.6 ± 5.7 mmHg, *P* < 0.001). APP-assisted self-care patients were more likely to be female (53.2% vs. 49.7, *P* = 0.018), unmarried (12.7% vs. 9.0%, *P* < 0.001), native resident (97.3% vs. 91.0%, *P* < 0.001), urban citizen (99.8% vs. 99.1%, *P* < 0.001). Patients with APP-assisted also had more chronic diseases, including diabetes (42.5% vs. 37.4%, *P* < 0.001), coronary heart disease (34.4% vs. 28.7%, *P* < 0.001), and stroke (16.7% vs. 14.4%, *P* = 0.039) than those patients without.

**Table 1:** Baseline information of hypertensive patients with and without APP-assisted self-care (*N* = 5937)

	Total (*N* = 5937)	With APP-assisted self-care (*N* = 4454)	Without APP-assisted self-care (*N* = 1483)	*P* value
Age, y	66.2 ± 10.8	66.5 ± 10.2	65.5 ± 12.4	0.004
Gender, n (%)				0.018
Male	2829(47.6)	2083(46.8)	746(50.3)	
Female	3108(52.4)	2371(53.2)	737(49.7)	
Marriage status, n (%)				< 0.001
Married	5238(88.2)	3888(87.3)	1350(91.0)	
Single/divorced/widowed	699(11.8)	566(12.7)	133(9.0)	
Native resident, n (%)				< 0.001
No	250(4.2)	117(2.6)	133(9.0)	
Yes	5687(95.8)	4337(97.3)	1350(91.0)	
Population composition, n (%)				< 0.001
Urban	5914(99.6)	4444(99.8)	1470(99.1)	
Rural	23(0.4)	10(0.2)	13(0.9)	
Comorbidity, n (%)				
Diabetes	2447(41.2)	1892(42.5)	555(37.4)	0.001
Coronary artery disease	1995(33.6)	1530(34.4)	425(28.7)	< 0.001
Stroke	958(16.1)	744(16.7)	214(14.4)	0.039
Height,cm	163.6 ± 8.6	163.4 ± 8.6	164.5 ± 8.6	< 0.001
Weight, Kg	69.3 ± 11.7	69.3 ± 11.5	69.5 ± 12.0	0.565
Body Mass Index, kg/m^2^	25.8 ± 3.5	25.9 ± 3.5	25.6 ± 3.6	0.052
Waist, cm	88.5 ± 9.5	88.8 ± 9.5	87.6 ± 9.2	< 0.001
Hip, cm	98.0 ± 9.2	98.4 ± 9.2	96.9 ± 9.2	< 0.001
Waist-hip ratio	0.9 ± 0.1	0.9 ± 0.1	0.90 ± 0.1	0.300
Fasting blood-glucose	6.3 ± 1.0	6.3 ± 1.1	6.3 ± 0.9	0.117
Total cholesterol	4.9 ± 1.0	4.8 ± 1.0	4.9 ± 0.9	0.589
Systolic BP, mmHg				
Baseline	128.7 ± 7.2	128.0 ± 7.3	130.8 ± 6.6	< 0.001
Follow-up	124.1 ± 13.8	122.6 ± 13.6	128.5 ± 13.5	< 0.001
Difference	-4.6 ± 15.6	-5.4 ± 15.5	-2.3 ± 15.9	< 0.001
Diastolic BP, mmHg				
Baseline	77.0 ± 5.7	76.8 ± 5.6	77.6 ± 5.7	< 0.001
Follow-up	73.5 ± 10.6	72.6 ± 10.7	76.2 ± 9.5	< 0.001
Difference	-3.5 ± 11.9	-4.2 ± 12.0	-1.3 ± 11.1	< 0.001
Follow-up BP control, n (%)				
Systolic	3886(65.4)	3000(67.4)	886(59.7)	< 0.001
Diastolic	4720(79.5)	3596(80.7)	1124(75.8)	< 0.001
Overall	3661(61.6)	2827(63.4)	834(56.2)	< 0.001

*Abbreviation*: Data are mean ± standard deviation except where indicated otherwise. *APP* application. *BP* blood pressure

### Detecting predictors for BP control levels

Compared with baseline, the community management resulted in mean SBP and DBP reductions of 4.6 mmHg and 3.5 mmHg at follow-up, respectively. The effect of hypertension management on decreases in SBP and DBP in follow-up compared with baseline was analyzed and subgroup analyses were conducted based on demographic characteristic, comorbidities, baseline date, APP-assisted self-care, antihypertensive medication adherence and follow-up time (Table 2).

**Table 2:** The effect of hypertension management on SBP and DBP level

	Patients	Difference in SBP, mmHg					Difference in DBP, mmHg				
		Mean (95% CI)	B	*P* value	B^a0^	*P* value*	Mean (95% CI)	B	P value	B^a^	*P* value*
Age (years), n (%)			-2.30	< 0.001	-0.53	0.124		-2.09	0.497	-0.34	0.215
≤ 65	2902(48.8)	-3.42(-3.97,-2.88)					-3.36(-3.77,-2.95)				
> 65	3035(51.2)	-5.73(-6.28,-5.14)					-3.57(-4.01,-3.05)				
Male, n (%)			0.36	0.379	0.61	0.081		0.04	0.904	0.30	0.276
No	3108(52.4)	-4.77(-5.35,-4.28)					-3.49(-3.93,-3.07)				
Yes	2829(47.6)	-4.41(-4.97,-3.86)					-3.45(-3.90,-3.06)				
Marriage status, n (%)			0.94	0.133	-0.41	0.428		-0.17	0.719	-0.17	0.675
Married	5238(88.2)	-4.49(-4.95,-4.08)					-3.49(-3.82,-3.17)				
Single/divorced/widowed	699(11.8)	-5.43(-6.56,-4.34)					-3.32(-4.19,-2.42)				
Native resident, n (%)			1.04	0.303	-0.16	0.847		0.57	0.456	-0.35	0.870
No	5687(95.8)	-4.65(-5.07,-4.24)					-3.50(-3.83,-3.18)				
Yes	250(4.2)	-3.60(-5.30,-1.97)					-2.92(-4.10,-1.74)				
Population composition, n (%)			-1.93	0.555	-2.01	0.453		0.65	0.794	-0.72	0.291
Urban	5914(99.6)	-4.59(-5.00,-4.19)					-3.47(-3.77,-3.20)				
Rural	23(0.4)	-6.52(-13.25,1.57)					-2.83(-8.93,2.81)				
Obesity, n (%)			-0.56	0.196	0.02	0.965		-0.25	0.452	0.01	0.993
No	4028(67.8)	-4.42(-4.93,-3.92)					-3.39(-3.77,-3.02)				
Yes	1909(32.2)	-4.98(-5.68,-4.31)					-3.64(-4.15,-3.09)				
Increased Waist-hip ratio, n (%)			-0.83	0.046	-0.37	0.289		-0.74	0.018	-0.40	0.146
No	3578(60.2)	-4.27(-4.77,-3.78)					-3.18(-3.55,-2.81)				
Yes	2359(39.8)	-5.10(-5.71,-4.48)					-3.92(-4.39,-3.42)				
Diabetes, n (%)			-1.29	0.002	-1.52	< 0.001		-0.91	0.004	-1.06	< 0.001
No	3490(58.8)	-4.07(-4.59,-3.56)					-3.10(-3.47,-2.69)				
Yes	2447(41.2)	-5.36(-6.02,-4.77)					-4.01(-4.54,-3.54)				
Coronary artery disease, n (%)			-0.05	0.909	0.34	0.342		0.35	0.285	0.39	0.176
No	3982(67.0)	-4.59(-5.07,-4.08)					-3.59(-3.97,-3.23)				
Yes	1955(33.0)	-4.63(-5.35,-3.93)					-3.24(-3.81,-2.69)				
Stroke, n (%)			-1.16	0.035	-0.12	0.784		-0.01	0.993	0.12	0.741
No	4979(83.8)	-4.41(-4.89,-4.01)					-3.47(-3.79,-3.16)				
Yes	958(16.2)	-5.57(-6.49,-4.74)					-3.47(-4.22,-2.73)				
Baseline date, n (%)			-3.39	< 0.001	-2.91	< 0.001		-2.44	< 0.001	-2.39	< 0.001
2014–2017	3822(64.4)	-4.05(-4.52,-3.68)					-3.05(-3.38,-2.72)				
2018–2021	2115(35.6)	-5.50(-5.98,-5.03)					-3.75(-4.21,-3.29)				
APP-assisted self-care, n (%)			-3.11	< 0.001	-4.59	< 0.001		-2.88	< 0.001	-2.91	< 0.001
No	1483(25.0)	-2.27(-3.12,-1.49)					-1.31(-1.89,-0.72)				
Yes	4454(75.0)	-5.38(-5.86,-4.94)					-4.19(-4.59,-3.84)				
Antihypertensive medication adherence, n (%)			-13.47	< 0.001	-13.04	< 0.001		-6.95	< 0.001	-6.86	< 0.001
No	846(14.2)	6.95(5.82,8.14)					2.49(1.68,3.26)				
Yes	5091(85.8)	-6.52(-6.96,-6.12)					-4.46(-4.78,-4.15)				
Follow-up time, n (%)			-2.04	< 0.001	0.55	0.219		-1.15	0.001	0.68	0.057
≤ 1 year	1443(24.4)	-3.05(-3.74,-2.27)					-2.60(-3.13,-2.05)				
> 1 year	4494(75.6)	-5.10(-5.57,-4.64)					-3.75(-4.10,-3.36)				

*Abbreviation*: *SBP* Systolic blood pressure, *DBP* Diastolic blood pressure, *B* Beta coefficient

B^a^, adjusted beta coefficient

^*^ Adjusted *P* values

Univariate GLM analysis using the difference in SBP or DBP level between baseline and follow-up as the dependent variable reveled that age (B = -2.30, *P* < 0.001), increased waist-hip ratio (B = -0.83, *P* = 0.046), diabetes (B = -1.29, *P* = 0.002), stroke (B = -1.16, *P* = 0.035), baseline date (B = -3.39, *P* < 0.001), APP-assisted self-care (B = -3.11, *P* < 0.001), antihypertensive medication adherence (B = -13.47, *P* < 0.001) and follow-up time (B = -2.04, *P* < 0.001) was associated with decreases in SBP level; increased waist-hip ratio (B = -0.74,*P* = 0.018), diabetes (B = -0.91, *P* = 0.004), baseline date (B = -2.44, *P* < 0.001), APP-assisted self-care (B = -2.88, *P* < 0.001), antihypertensive medication adherence (B = -6.95, *P* < 0.001) and follow-up time (B = -1.15, *P* = 0.001) was associated with decreases in DBP level.

After adjusted covariates in the multivariable GLM analysis, the results revealed that patients with diabetic exhibited a significant SBP reduction (B = -1.52, *p* < 0.001; -5.36 mmHg, 95% CI [-6.02, -4.77] vs. -4.07 mmHg, 95%CI [-4.59, -3.56]) and DBP reduction (B = -1.06, *p* < 0.001; -4.016 mmHg, 95% CI [-4.54, -3.54] vs. -3.10 mmHg, 95%CI [-3.47, -2.69]) compared to those without diabetic. Later baseline date was associated with significant lower SBP reduction (B = -2.91, *p* < 0.001; -5,50 mmHg, 95% CI [-5.98, -5.03] vs. -4.05 mmHg, 95%CI [-4.52, -3.68]) and DBP reduction (B = -2.39, *p* < 0.001; -3,75 mmHg, 95% CI [-4.21, -3.29] vs. -3.05 mmHg, 95%CI [-3.38, -2.72]). In those patients with APP-assisted self-care, the results suggested a significant reduction both in SBP level (B = -4.59, p < 0.001; -5.38 mmHg, 95% CI [-5.86, -4.94] vs. -2.27 mmHg, 95%CI [-3.12, -1.49]) and in DBP level (B = -2.91, p < 0.001; -4.19 mmHg, 95% CI [-4.59, -3.84] vs. -1.31 mmHg, 95%CI [-1.89, -0.72]). Similarly, for patients with antihypertensive medication adherence, significant reduction was also found in SBP level (B = -13.04, *p* < 0.001; -6.52 mmHg, 95% CI [-6.96, -6.12] vs. 6.95 mmHg, 95%CI [5.82,8.14]) and in DBP level (B = -6.86, *p* < 0.001; -4.46 mmHg, 95% CI [-4.78, -4.15] vs. 2.49 mmHg, 95%CI [1.68,3.26]).

### Detecting predictors for BP control rates

There were 3661(61.6%) hypertension patients with BP control, which increased from 55.0% in 2014 to 75.0% in 2021 (Supplement Fig. 2). The effect of hypertension management on overall BP control in follow-up was analyzed and subgroup analyses were conducted (Table 3). Univariate GLM analysis using poor BP control as the dependent variable reveled that native resident (*P* < 0.001), diabetes (*P* < 0.001), coronary artery disease (*P* < 0.001), stroke (*P* = 0.001), baseline date (*P* < 0.001), APP-assisted self-care (*P* < 0.001), antihypertensive medication adherence (*P* < 0.001), and follow-up time (*P* < 0.001) are important factors associated with poor BP control. After adjusted covariates in the multivariable GLM analysis, the results revealed that native resident (OR = 0.50, 95%CI [0.34,0.74], *P* < 0.001), diabetes (OR = 1.50, 95%CI [1.33,1.70], *P* < 0.001), coronary artery disease (OR = 1.35, 95%CI [1.19,1.53], *P* < 0.001), stroke (OR = 1.20, 95%CI [1.02,1.41], *P* = 0.026), baseline date (OR = 0.72, 95%CI [0.62,0.83], *P* < 0.001), APP-assisted self-care (OR = 0.51,95%CI [0.44,0.59], *P* < 0.001), antihypertensive medication adherence (OR = 0.14,95%CI [0.11,0.16], *P* < 0.001) and follow-up time (OR = 2.02,95% CI [1.70,2.40], *P* < 0.001) are predictors for BP control.

**Fig. 2:**
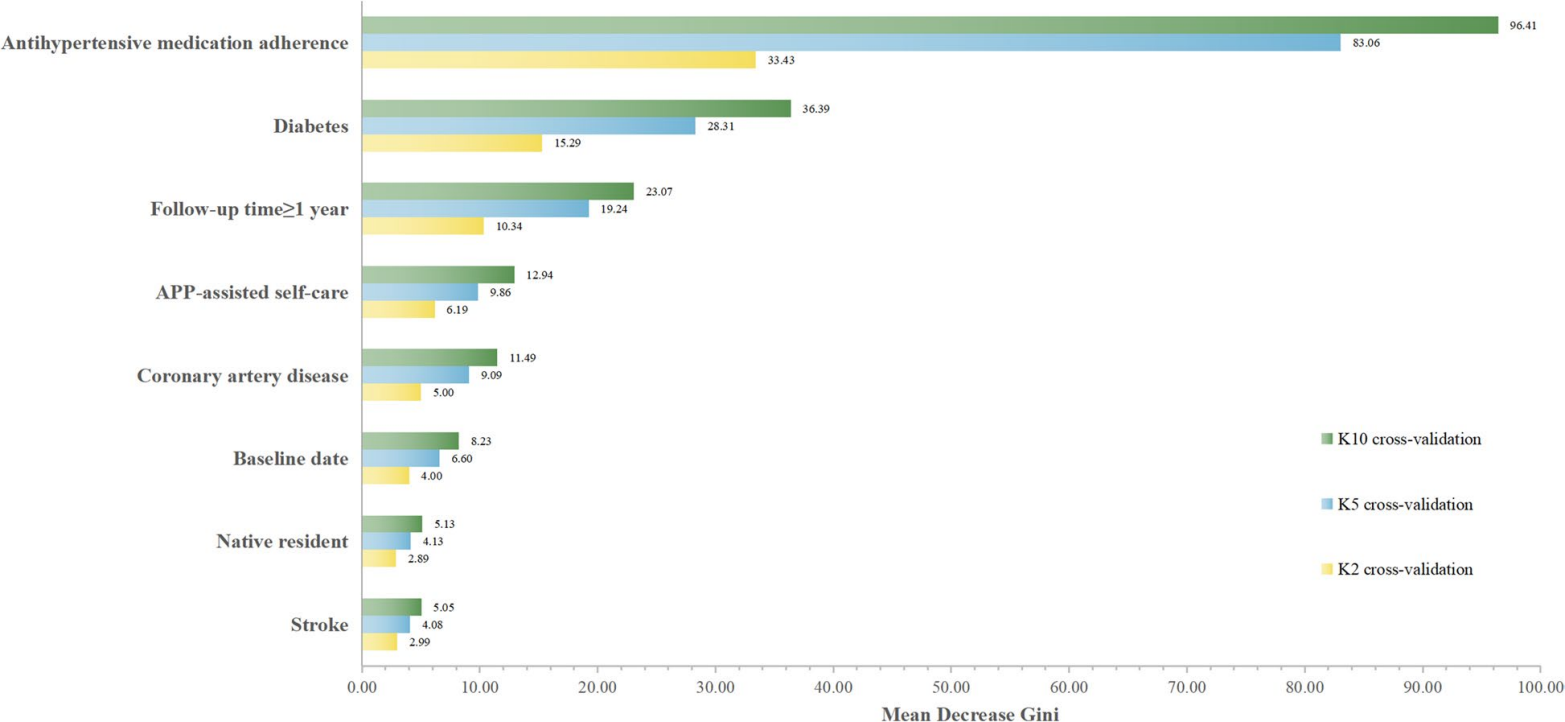
The importance of predictors for poor BP control in random forest algorithm

**Table 3:** Overall BP control in hypertension patients with different characteristics

	Total (*N* = 5937)	Good control (*N* = 3661)	Poor control (*N* = 2276)	Univariate analysis		Multivariable analysis	
				OR(95%CI)	*P* value	OR(95%CI)	*P* value
Age (years), n (%)				1.06(0.96,1.18)	0.251	0.89(0.78, 1.02)	0.091
≤ 65	2902(48.8)	1811(49.5)	1091(47.9)				
> 65	3035(51.2)	1850(50.5)	1185(52.1)				
Male, n(%)				1.03(0.93,1.14)	0.612	0.99(0.87, 1.13)	0.893
No	3108(52.4)	1926(52.6)	1182(51.9)				
Yes	2829(47.6)	1735(47.4)	1094(48.1)				
Marriage status, n (%)				0.92(0.78,1.08)	0.281	1.00(0.83, 1.21)	0.986
Married	5238(88.2)	3243(88.6)	1995(87.7)				
Single/divorced/widowed	699(11.8)	418(11.4)	281(12.3)				
Native resident, n (%)				0.44(0.32, 0.60)	< 0.001	0.50(0.34, 0.74)	< 0.001
No	250(4.2)	195(5.3)	55(2.4)				
Yes	5687(95.8)	3466(94.7)	2221(97.6)				
Population composition, n (%)				1.03(0.45,2.39)	0.937	1.76(0.62, 4.97)	0.285
Urban	5914(99.6)	3647(99.6)	2267(99.6)				
Rural	23(0.4)	14(0.4)	9(0.4)				
Obesity, n (%)				1.09(0.98, 1.22)	0.121	0.98(0.86, 1.11)	0.724
No	4028(67.8)	2511(68.6)	1517(66.6)				
Yes	1909(32.2)	1150(31.4)	759(33.4)				
Increased Waist-hip ratio, n (%)				1.09(0.98,1.20)	0.131	1.07(0.94, 1.22)	0.279
No	3578(60.2)	2234(61.0)	1344(59.1)				
Yes	2359(39.8)	1427(39.0)	932(40.9)				
Diabetes, n (%)				1.91(1.71,2.12)	< 0.001	1.50(1.33, 1.70)	< 0.001
No	3490(58.8)	2372(64.8)	1118(49.1)				
Yes	2447(41.2)	1289(35.2)	1158(50.9)				
Coronary artery disease, n (%)				1.54(1.38, 1.71)	< 0.001	1.35(1.19, 1.53)	< 0.001
No	3982(67.0)	2590(70.7)	1392(61.2)				
Yes	1955(33.0)	1071(29.3)	884(38.8)				
Stroke, n (%)				1.27(1.11, 1.46)	0.001	1.20(1.02, 1.41)	0.026
No	4979(83.8)	3117(85.1)	1862(81.8)				
Yes	958(16.2)	544(14.9)	414(18.2)				
Baseline date, n(%)				0.63(0.56–0.70)	< 0.001	0.72(0.62, 0.83)	< 0.001
2014–2017	3822(64.4)	2210(60.4)	1612(70.8)				
2018–2021	2115(35.6)	1451(39.6)	664 (29.2)				
APP-assisted self-care, n (%)				0.74(0.66–0.83)	< 0.001	0.51(0.44, 0.59)	< 0.001
No	1483(25.0)	834(22.8)	649(28.5)				
Yes	4454(75.0)	2827(77.2)	1627(71.5)				
A*ntihypertensive* medication adherence, n (%)				0.16(0.14–0.20)	< 0.001	0.14(0.11, 0.16)	< 0.001
No	846(14.2)	218(6.0)	628(27.6)				
Yes	5091(85.8)	3443(94.0)	1648 (72.4)				
Follow-up time, n (%)				1.96(1.72–2.24)	< 0.001	2.02(1.70, 2.40)	< 0.001
≤ 1 year	1443(24.4)	1055(28.8)	388(17.0)				
> 1 year	4494(75.6)	2606(71.2)	1888(83.0)				

### Testing prediction accuracy of predictors in machine learning algorithm

Multivariate GLM analysis implicated eight independent variables that were significantly associated with poor BP control: native resident, diabetes, coronary artery disease, stroke, baseline date, APP-assisted self-care, antihypertensive medication adherence, and follow-up time. All independent variables were then incorporated into the machine learning system, generating a predictive model of poor BP control. Te compare the performance of four algorithms were shown in Table 4 for twofold, fivefold and tenfold cross-validations. It is observed that RF-based algorithm performs better for all cross-validations compared to DT, SVB, and NB, giving the highest classification accuracy and area under the curve (AUC). Moreover, other performance parameters such as sensitivity (SE), specificity(SPE), positive predictive value(PPV), negative predictive value(NPV) for three cross-validations were also shown in Table 4. The DT was drawn to identify important factors associated with BP control (Supplement Fig. 3). Mean Decrease Gini (MDG) was calculated to rank the important predictors with poor BP control in the RF algorithm. A twofold, fivefold and tenfold cross-validation was then performed within the train set. The mean values of MDG from cross-validated results were shown in Fig. 2.

**Table 4:** Performance evaluation of RF and other machine learning algorithm

Cross-validation	Algorithm	Performance evaluation parameters					
		ACC(%)	SE(%)	SPE(%)	PPV (%)	NPV (%)	AUC
K2	DT	70.12	42.10	87.52	67.66	70.86	0.6712
	SVM	70.95	44.36	87.46	68.75	71.66	0.6642
	NB	66.33	45.00	79.60	57.83	69.95	0.6874
	RF	71.03	34.88	91.01	70.88	69.20	0.7291
K5	DT	70.14	42.09	87.50	67.57	70.89	0.6788
	SVM	70.96	44.46	87.46	68.77	71.71	0.6601
	NB	66.32	44.99	79.56	57.79	69.93	0.6885
	RF	71.96	35.35	91.03	71.45	69.31	0.7316
K10	DT	70.11	42.11	87.56	67.76	70.90	0.6686
	SVM	70.94	44.45	87.43	68.78	71.68	0.6619
	NB	70.92	44.40	87.46	68.83	71.64	0.6774
	RF	71.37	33.53	91.63	71.34	68.91	0.7286

*Abbreviation*: *DT* Decision tree, *SVM* Support Vector Machine, *NB* Naïve Bayes, *RF* Random forest, *ACC* Accuracy, *SE* Sensitivity, *SPE* Specificity, *PPV* Positive predictive value, *NPV* Negative predictive value, *AUC* Area under the curve, *K2* Twofold cross-validation, *K5* Fivefold cross-validation, *K10* Tenfold cross-validation

## Discussion

This community-based cohort study was to examine the effectiveness of community healthcare in controlling BP and to investigate the role of mobile APP-assisted self-care. Compared with baseline, the community management resulted in mean SBP and DBP reductions of 4.6 mmHg and 3.5 mmHg. There were 61.6% hypertensive patients with good BP control, increasing from 55.0% in 2014 to 75.0% in 2021. After adjusting for covariates, common predictors in GLM models and machine learning algorithm revealed that antihypertensive medication adherence, diabetes, and APP-assisted self-care were associated with BP control. Overall, the APP-assisted self-care would be helpful for the management of hypertensive patients in a Beijing community.

The results of this study were similar to other cohort studies, confirmed the importance of compliance management in the antihypertensive treatment [19, 20], and the unsatisfied hypertension control in treated patients with chronic disease history such as diabetes [20, 21], coronary artery disease [20, 21], and stroke [21]. Moreover, our results further confirmed the effect of APP-assisted self-care model with the guidance of general practitioners when compared to previous studies reporting self-management on hypertensive patients in other counties [9–13]. One strength of our study is the large population-based sample in a Chinese community. To date, only a few studies [14–16] concerning self-management for BP control have been reported in China, but most of them were small sample size, investigator initiated cross-sectional survey [14] or intervention study [15], which are insufficient to address the huge burden of hypertension.

In recent years, community-based family-doctor-contracted services have been put into practice in many cities in China, such as Shanghai [22] and Beijing [17]. Research in Shanghai [22] revealed the self-management might help to achieve greater control of noncommunicable diseases. The advantage of IFOCM program in Beijing is that it initiated by a government for long-term continuous and comprehensive management of hypertensive patients [17]. This patient-centered healthcare model similar to home-based primary care practices reported in the United States [23–25]. In the IFOCM, hypertensive patient needs to be provided periodic follow-up and continuity of BP management, which is essential to improve hypertension awareness, treatment and control. The emergence of mobile health makes it more convenient for community health service to provide healthcare and disease management. The advantage of APP in IFOCM program is that patients can easily access to APP from the community WeChat official account, without download from any other APP stores and regular update. On the online platform, patients can check own health record,medical report,physiological data such as BP level, glucose level and body weight, and interactive with contracted family doctor (Fig. 1). In the present cohort study, we provided a mobile APP-assisted self-care model for community hypertension patients in Beijing, China, which lowered patients’ BP level and improve the BP control rate at follow-up. This model could serve as a good example for managing hypertensive patients registered in other community health services, especially in those with limited family doctors or general practitioners. Future work should apply applications of mobile Health for the control of other chronic diseases in community settings.

This study had several limitations. First, there were 50.7% of baseline hypertensive patients not participated in IFOCM program and failed to attend the follow-up survey in the present study. Therefore, a selection bias may exist. Second, although we adjusted all measured covariates when performing analysis, there were still unmeasured covariates, such as the number of antihypertensive drugs, what kind of drug prescribed to BP control, the primary IFOCM system could not access to outpatient records for the present, thus confounding bias cannot be avoided in the statistic analysis. Third, this was a single center study in community health service, multicenter study with long-term follow-up is needed to further examine the effect of App-assisted self-care in community hypertension management.

## Conclusion

In summary, this study found that antihypertensive medication adherence, diabetes, and APP-assisted self-care were associated with follow-up BP level and BP control rate. The APP-assisted self-care succeeded in the management of hypertensive patients in a Beijing community.

## Supplementary Information

**Additional file 1:** **SFigure 1** Study flow chart.

**Additional file 2:** **SFigure 2** Overall BP control rate of new diagnosed cases in community.

**Additional file 3:** **SFigure 3** Predictive decision tree model of poor BP control in hypertensive patients

**Additional file 4:** **Stable.** Baseline information of hypertensive patients participated or not participated in the IFOCM program (*N=*12050).

## Data Availability

The datasets generated and/or analysed during the current study are not publicly available due to local policy but are available from the corresponding author on reasonable request.

## Abbreviations

IFOCM: Intelligent family physician-optimised collaborative model
BP: Blood pressure
SBP: Systolic blood pressure
DBP: Diastolic blood pressure
BMI: Body mass index
RF: Random forest
DT: Decision tree
SVM: Support vector machine
NB: Naïve Bayes
SD: Standard deviation
OR: Odds ratio
GLM: Generalized linear model
MDG: Mean Decrease Gini
AUC: Area under the curve
SE: Sensitivity
SPE: Specificity
PPV: Positive predictive value
NPV: Negative predictive value
