# Supplementary figures and images for: Mobile APP-assisted family physician program for improving blood pressure outcome in hypertensive patients
Source: BMC Prim Care. 2023 Jan 10;24:8. doi: 10.1186/s12875-023-01965-2 (PMC9832760; doi:10.1186/s12875-023-01965-2)

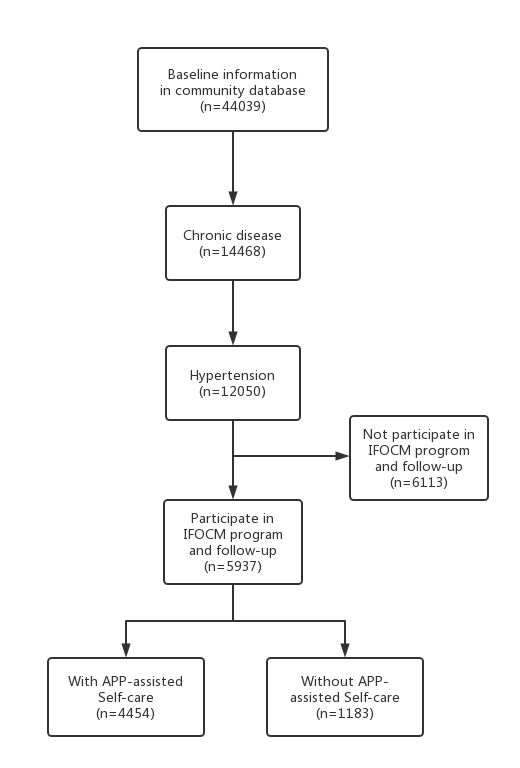

Supplement: Supplementary file 1 — Additional file 1: SFigure 1 Study flow chart. [file 12875_2023_1965_MOESM1_ESM.jpg]

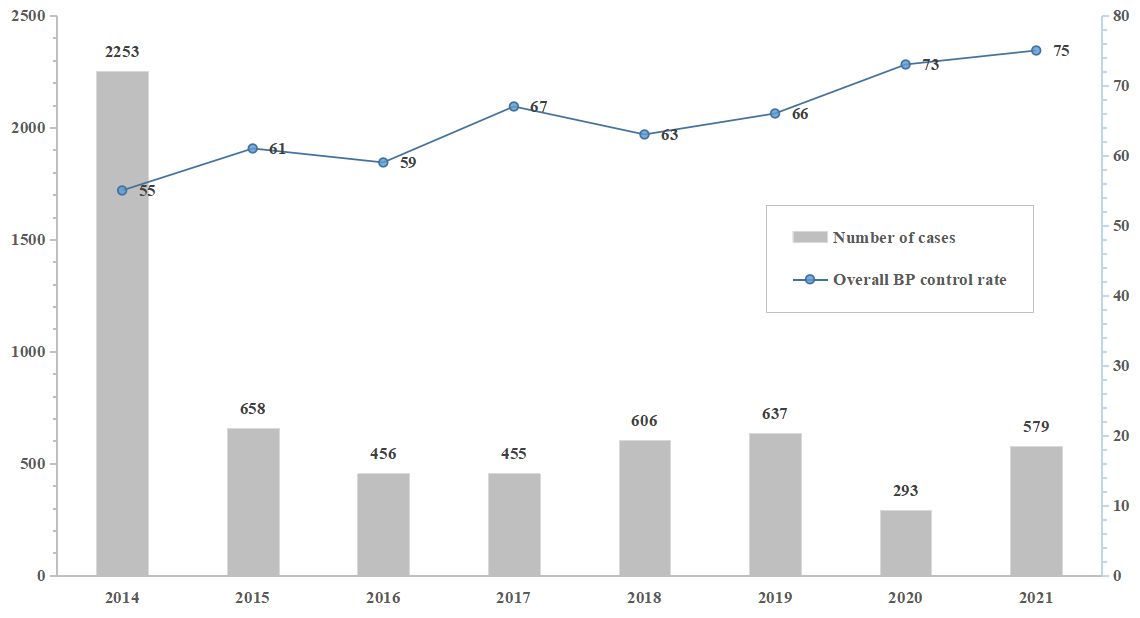

Supplement: Supplementary file 2 — Additional file 2: SFigure 2 Overall BP control rate of new diagnosed cases in community. [file 12875_2023_1965_MOESM2_ESM.jpg]

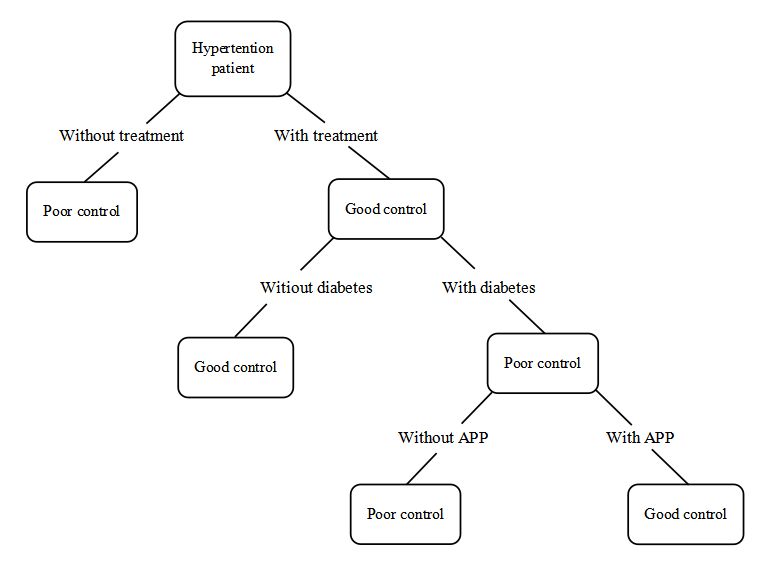

Supplement: Supplementary file 3 — Additional file 3: SFigure 3 Predictive decision tree model of poor BP control in hypertensive patients [file 12875_2023_1965_MOESM3_ESM.jpg]
