# Supplementary material for: Mobile APP-assisted family physician program for improving blood pressure outcome in hypertensive patients
Source: BMC Prim Care. 2023 Jan 10;24:8. doi: 10.1186/s12875-023-01965-2 (PMC9832760; doi:10.1186/s12875-023-01965-2)
Supplement: Supplementary file 4 — Additional file 4: Stable. Baseline information of hypertensive patients participated or not participated in the IFOCM program (N=12050). [file 12875_2023_1965_MOESM4_ESM.docx]

**STable. Baseline information of hypertensive patients participated or not participated in the IFOCM program (N=12050)**

|  | With Signed family physician  (N=5937) | Without sighed family physician  (N=6113) | P value |
| --- | --- | --- | --- |
| Age, y | 66.2±10.8 | 65.9±10.8 | 0.086 |
| Gender, n(%) |  |  | 0.306 |
| Male | 2829(47.6) | 2856(46.7) |  |
| Female | 3108(52.4) | 3257(53.3) |  |
| Marriage status, n(%) |  |  | 0.185 |
| Married, n(%) | 5238(88.2) | 5345(87.4) |  |
| Single/divorced/widowed | 699(11.8) | 768(12.6) |  |
| Native resident , n(%) |  |  | <0.001 |
| No | 5687(95.8) | 5710(93.4) |  |
| Yes | 250(4.2) | 403(6.6) |  |
| Population composition, n(%) |  |  | <0.001 |
| Urban | 5914(99.6) | 6032(98.7) |  |
| Rural | 23(0.4) | 81(1.3) |  |
| Comorbidity, n(%) |  |  |  |
| Diabetes | 2447(41.2) | 2457(40.2) | 0.253 |
| Coronary artery disease | 1995(33.6) | 2686(43.9) | <0.001 |
| Stroke | 958(16.1) | 1104(18.0) | 0.005 |
| Height,cm | 163.6±8.60 | 164.3±8.12 | <0.001 |
| Weight, Kg | 69.3±11.6 | 68.9±11.7 | 0.039 |
| Body Mass Index, kg/m^2^ | 25.8±3.49 | 25.4±3.52 | <0.001 |
| Waist, cm | 88.5±9.5 | 87.9±9.8 | <0.001 |
| Hip, cm | 96.8±9.3 | 98.0±9.2 | <0.001 |
| Waist-hip ratio | 0.89±0.1 | 0.90±0.1 | <0.001 |
| Systolic blood pressure | 128.6±7.2 | 129.8±8.0 | <0.001 |
| Diastolic blood pressure | 75.8±6.3 | 76.9±5.7 | <0.001 |
| Fasting blood-glucose | 6.3±1.0 | 6.2±1.3 | <0.001 |
| Total cholesterol | 5.0±1.0 | 4.9±0.9 | 0.052 |
